# Diffusion weighted imaging and diffusion kurtosis imaging in abdominal oncological setting: why and when

**DOI:** 10.1186/s13027-022-00441-3

**Published:** 2022-06-09

**Authors:** Vincenza Granata, Roberta Fusco, Andrea Belli, Ginevra Danti, Eleonora Bicci, Carmen Cutolo, Antonella Petrillo, Francesco Izzo

**Affiliations:** 1 Division of Radiology, “Istituto Nazionale Tumori IRCCS Fondazione Pascale – IRCCS di Napoli”, I-80131 Naples, Italy; 2 Medical Oncology Division, Igea Spa, Naples, Italy; 3 Division of Hepatobiliary Surgical Oncology, “Istituto Nazionale Tumori IRCCS Fondazione Pascale – IRCCS di Napoli”, I-80131 Naples, Italy; 4 Department of Radiology, Azienda Ospedaliero-Universitaria Careggi, Florence, Italy; 5 Italian Society of Medical and Interventional Radiology, SIRM Foundation, Milan, Italy; 6 Department of Medicine, Surgery and Dentistry, University of Salerno, Salerno, Italy

**Keywords:** Magnetic resonance imaging, DWI, DKI, Oncological setting

## Abstract

This article provides an overview of diffusion kurtosis (DKI) imaging in abdominal oncology. DKI allows for more data on tissue structures than the conventional diffusion model (DWI). However, DKI requires high quality images at *b*-values greater than 1000 s/mm^2^ and high signal-to-noise ratio (SNR) that traditionally MRI systems are not able to acquire and therefore there are generally amplified anatomical distortions on the images due to less homogeneity of the field. Advances in both hardware and software on modern MRI scanners have currently enabled ultra-high *b*-value imaging and offered the ability to apply DKI to multiple extracranial sites. Previous studies have evaluated the ability of DKI to characterize and discriminate tumor grade compared to conventional DWI. Additionally, in several studies the DKI sequences used were based on planar echo (EPI) acquisition, which is susceptible to motion, metal and air artefacts and prone to low SNRs and distortions, leading to low quality images for some small lesions, which may affect the accuracy of the results. Another problem is the optimal *b*-value of DKI, which remains to be explored and not yet standardized, as well as the manual selection of the ROI, which could affect the accuracy of some parameters.

## Introduction

Diffusion-Weighted Imaging (DWI) has been recognized as a significant magnetic resonance imaging (MRI) tool for disease assessment primarily in oncology [1–13]. The intensity, direction and time profile of the imaging gradient affect the diffusion sensitivity of water molecules and in DWI are included in a single simplified parameter called *b*-value (unit: s/mm^2^) [14–18]. The images acquired with different *b*-values were processed to obtain a parametric map that allows the quantification of the of the apparent diffusion coefficient that is linked to the microscopic mobility of water. In clinical setting, DWI is performed using *b*-values up to 800–1000 s/mm^2^, and the map quantification is performed using a monoexponential model considering that the diffusion water mobility follows a normal Gaussian model and then that the diffusion behaviour results in linear decay of the natural logarithm of the DWI signal intensity (SI) as the *b*-value increases and the slope represents the apparent diffusion coefficient (ADC) [19–24]. However, it is known that the water molecules diffusion within tissue follows a non-Gaussian model and for this reason in 2005 Jensen et al. [25] described a non-Gaussian approach, named Diffusion Kurtosis imaging (DKI) to assess tissue water diffusion coefficients. By means of the DKI is possible to calculate the kurtosis median coefficient (MK), which assesses the variation of diffusion behaviour by a Gaussian, to a non-Gaussian model, and the diffusion coefficient (MD), which assesses the correction of the non-Gaussian bias [25–27]. The term dimensionless kurtosis describes the degree of deviation from the Gaussian distribution of the spin displacements along the observation axis and therefore, when the average over all directions is calculated, the mean kurtosis is obtained. The MD value provides novel diffusion properties that describe the tissue microstructure.

DKI allows for more data on tissue structures than the conventional diffusion model (DWI). However, DKI requires high quality images at *b*-values greater than 1000 s/mm^2^ and high signal-to-noise ratio (SNR) that traditionally MRI systems are not able to acquire and therefore there are generally amplified anatomical distortions on the images due to less homogeneity of the field. Advances in both hardware and software on modern MRI scanners have currently enabled ultra-high *b*-value imaging and offered the ability to apply DKI to multiple extracranial sites [28–44].

Hence, radiologists could benefit through a better understanding of the major concepts of DKI.

In this paper, we evaluate the basic principles of DKI and clinical applications in oncological setting within the recent peer-reviewed literature.

### Diffusion analysis: basic principles

DKI analyses non-Gaussian water diffusivity using a polynomial approach according to the following equation:

$${\text{S}}_{{\text{i}}} = {\text{S}}_{0} *{\text{e}}\left( { - {\text{b}}_{{\text{i}}} *{\text{D}}_{{{\text{app}}}} + 1/6 + {\text{b}}_{{\text{i}}}^{2} *{\text{D}}_{{{\text{app}}}}^{2} *{\text{K}}_{{{\text{app}}}} } \right)$$

In this, there are two variables, Dapp and Kapp, while S_0_ is the basal signal with *b* value = 0.

Kapp is the apparent diffusional kurtosis, which reflects the higher distribution of the high tissue diffusivities that occurs in the setting of the non-Gaussian diffusion behaviour. Kapp is determined by the curvature of the SI decay away from the plot that would be predicted by a monoexponential model. The Dapp is the diffusion coefficient corrected to take into account the observed non-Gaussian behaviour and is determined by the slope of the SI decay diagram.

The DKI approach offers radiologists the possibility of obtaining information on the anisotropic characteristics of tissues not obtainable with conventional DWI [27].

Tissue ADC at *b*-values below 1000 s/mm^2^ has been recognized as an assessment primarily of the extracellular space [27]. Cell arrangement, cell size distribution, cell density, extracellular space viscosity, glandular structures, and membrane integrity are all variables that can affect the diffusion of water into the extracellular space. Hence, lower ADC values have often been attributed to higher cell density. In contrast, Kapp has been sized to exemplify the direct relationships of water molecules to cell membranes and intracellular complexes, although it is also influenced by other hard-to-separate extracellular parameters [27]. In fact, at the nanoscopic level, water is an inhomogeneous substance due to the polar nature of its molecule. Furthermore, water molecules could form 3D arrays in the presence of interfaces with charged materials such as polarized cell membranes or organelles or protein molecules, resulting in organization in layers with reduced diffusivity [27]. In this scenario, DKI values have a higher specificity to reveal water interactions within cell and tissue components [27].

### Acquisition consideration

DKI is acquired using a standard DWI sequence also using ultra high *b*-values. Conventional DWI should require acquiring only two *b*-values for ADC evaluation while DKI should require at least three different *b*-values since there is an additional variable (Kapp) within the mathematical model. The possibility of acquiring more than three *b*-values and at least two *b*-values above and below 1000 s/mm^2^ should be considered to help capture non-Gaussian behaviour [27].

To obtain accurate DKI values, it is critical that high *b*-values have adequate SNR. However, this, at higher *b*-values, is remarkably challenging in body imaging, given the faster signal decays and the penchant for employing faster sequences. Therefore, it may be necessary to reduce the spatial resolution or increase the number of signal averages to maintain SNR [27].

Today, MR systems do not habitually offer in-line DKI post-processing options. Therefore, separate post-processing software are needed. At least, DKI assessment should offer two maps (Dapp and Kapp). Dapp map is similar to ADC map. Reductions in Dapp are usually correlated with increases in Kapp, both indicating abnormal diffusion behaviour in similar anatomic sites. However, a visual analysis of the two maps could be diagnostically not sufficient. Instead, quantitative evaluation is suggested to fully harness the complementary role of kurtosis in distinguishing tissue pathology [27].

### Clinical studies

In oncological setting, the interpretation of images comprises different phases of patient management (diagnosis, staging, and assessment of treatment response) [45–54]. The spread in expertise in cancer and the opportunity to obtain a tailored treatment by choosing a proper approach, as well as the management of patients within a multidisciplinary team has increased the patient prognosis [55–69]. In this scenario, the possibility to use a diagnostic tool that evaluates cancer characteristics at microscopic level explains the reasons for the great attention on DWI [70–76].

By quantifying the non-Gaussianity of the diffusion distribution in DWI, Kapp is a comparatively direct measure of the heterogeneity of the tissue microstructure. Recent studies have reported that DKI can reflect microstructural conditions more accurately than conventional DWI [77, 78]. These promising results ignite an interest in DKI that may allow for more comprehensive tissue characterization than conventional DWI.

#### Liver

**Fig. 1:**
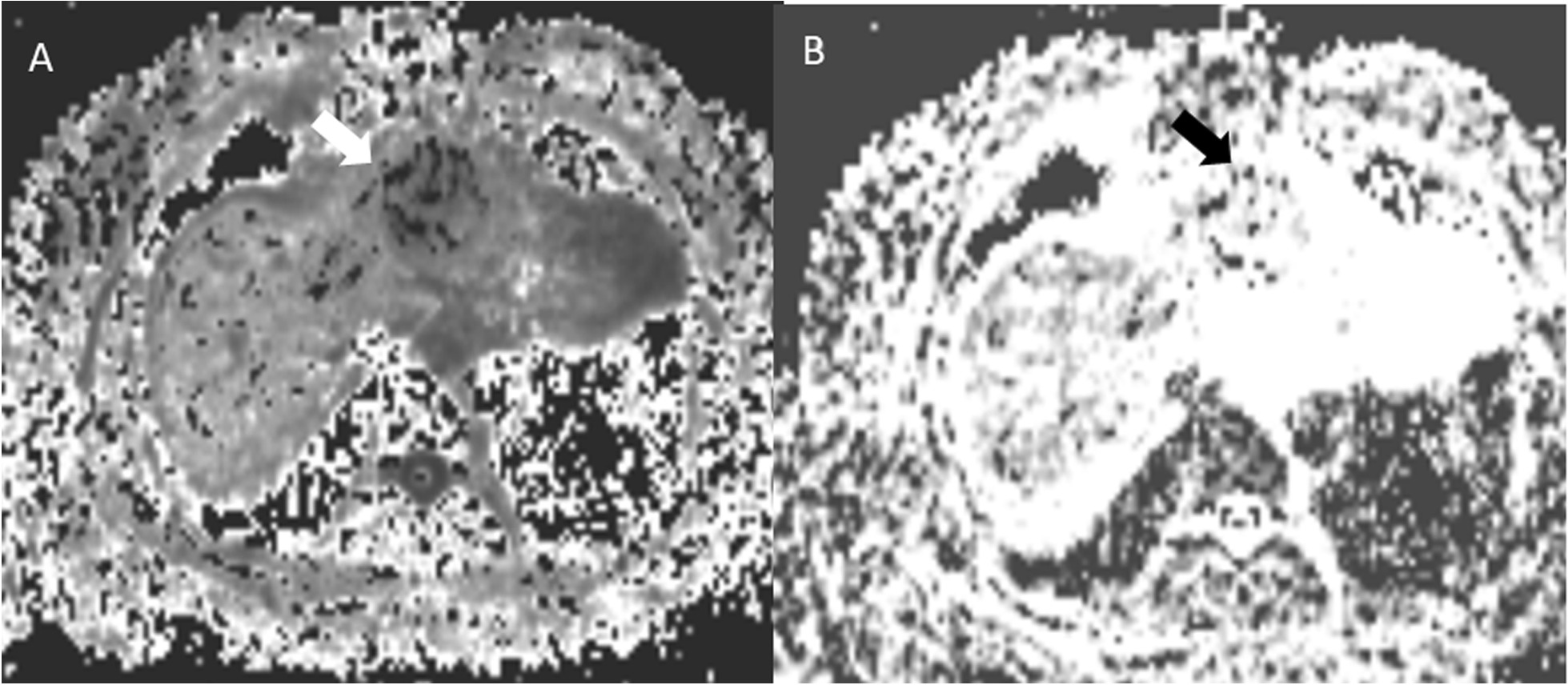
Woman 73 year with HCC on II hepatic segment. In **A** the mean coefficient of apparent kurtosis (MK) which showed the lesion with restricted diffusion and therefore hypointense, in **B** the mean diffusion coefficient (MD) which instead appeared hyperintense

Several researches have assessed the role of DKI in liver diseases, with particular regard in fibrosis and hepatocellular carcinoma (HCC), comprising different phases of patient management (Fig. 1).

Generally, hepatic fibrosis is represented as a reaction to a disorder associated with hepatocellular degeneration [79–84]. The amount of fibrous connective tissue increases due to the progression of inflammatory diseases with cellular degeneration or necrosis. During the process of fibrosis, molecules of collagen, glycosaminoglycans and proteoglycans are deposited in the extracellular space of the liver. Consequently, hepatic fibrosis accompanied by swelling of the hepatocytes and infiltration of inflammatory cells narrows the extracellular space, increasing the average value of kurtosis. Yoshimaru et al. [79] evaluated the relationship between DKI and liver function by comparing the mean value of kurtosis with the Child–Pugh score, the ALBI score and the ICG-R15 value, which are typical indicators of liver function [80] and demonstrating that liver function can be quantitatively assessed using the mean kurtosis value. Indeed, the fibrotic process could explain the positive correlation between the mean value of kurtosis and the Child–Pugh score or the ALBI score. However, there was little correlation between the mean value of kurtosis and the Child–Pugh score or the ALBI score presumably because the pattern of variation of the DKI and Child–Pugh scores or the ALBI scores differ according to the state of the hepatocyte tissue and liver function, respectively.

Recently, several studies have evaluated the role of DKI in HCC for the characterization and evaluation of the response to treatment [1, 83, 85–90]. However, the consistency and repeatability of the adapted parameters have not been assessed. It is known that more complex models with multiple parameters tend to oversize the data, resulting in poor repeatability and limited use in clinical practice. Furthermore, in order to capture the non-Gaussian diffusion behaviour of water molecules in biological tissues, maximum *b*-values of about 2000 s/mm^2^ have been proposed for the liver. A higher *b*-value means a lower SNR and less repeatability of the calculated parameters. Therefore, it is necessary to explore whether or not non-mono-exponential models may provide desirable repeatability of measurements for HCC. Rosenkrantz et al. [77] performed the DKI assessment in HCC using fresh liver explants. Twelve liver explants underwent MR study using a sequence with a maximum *b* value of 2000 s/mm^2^. A conventional mono-exponential model was used to calculate the ADC and a non-Gaussian model to evaluate Kapp and Dapp. They showed that 16 HCC had intermediate to substantial excess diffusional kurtosis and Dapp was 23% greater than ADC medium. ADC, Dapp, and Kapp had significant differences between responding and non- or partially responding lesions. Among the unresponsive nodules, cellularity showed a strong inverse association with ADC, a weaker inverse association with Dapp, and a direct association with Kapp [77].

With regard to prognosis, the pathological grade of HCC and microvascular invasion (MVI) are main involved features, since they are independent predictive features for recurrence and long-term survival after resection [91–94]. Cao et al. explored the performance of DKI in predicting the presence of microvascular invasion (MVI) and the histological grade of HCC and compared it to the conventional ADC value. The results of their study suggested that of all the diffusion parameters studied, MK might be the most promising factor in the systematic assessment of tumour biological behaviours and serve as an independent risk factor for early relapse after liver resection within one year [95]. Wang et al. [96] that showed the correlation between MK and histological grade of HCC have confirmed these results.

Few studies have evaluated DKI and liver metastases. Granata et al. assessed the role of DKI in patients with colorectal liver metastases to detect RAS mutation [97]. They showed a significant association between the group with RAS mutation and the group without RAS mutation with MK [MK standard deviation (STD)], MD, and the perfusion fraction (FP). The best results were reached by MK STD with an area under curve (AUC) of 0.80, an accuracy of 79% using a cut-off of 203.90 × 10^−3^ [97].

Ablative treatment is a minimally invasive approach that is usually used in the treatment of tumours [98–101]. Ablation treatment is believed as a potential first-line tool in small HCCs (< 3 cm) [101]. The goal of ablative treatment is necrosis. Therefore, tumour volume decrease may be absent with these treatments. Tumour features such as angiogenesis and hypoxia are more pertinent to assessing response, so as it is necessary to develop new functional biomarkers. Goshima et al. [87] assessed DKI and conventional DWI for evaluating treatment response in hypervascular HCC. Sixty-two patients (112 HCCs; viable, *n* = 63; non-viable, *n* = 49); underwent MRI; DKI was performed with different *b* values: 0, 100, 500, 1000, 1500, and 2000s/mm^2^. The MK and ADC values of the hepatic parenchyma and of the HCCs were assessed. The detectability of viable HCC based on MK and ADC was analysed. They also evaluated the correlation between Child–Pugh classes and MK or ADC values. The MK value was significantly higher for the viable lesions than for the non-viable lesions, while ADC values were significantly lower between the viable lesions and non-viable lesions. Considering that viable HCCs are characterized by structural complexity, with higher cellularity with nuclear atypia, more vascular hyperplasia or necrosis, and occasionally fatty deposition, it is known that DKI model represents better the complexity of biological tissues. However, it is essential that DKI might be evaluated in a reproducible manner and therefore is mandatory to standardize the protocol, establishing the strength and number of “*b*” values, the model to evaluate quantitative parameters [87].

#### Pancreas

**Fig. 2:**
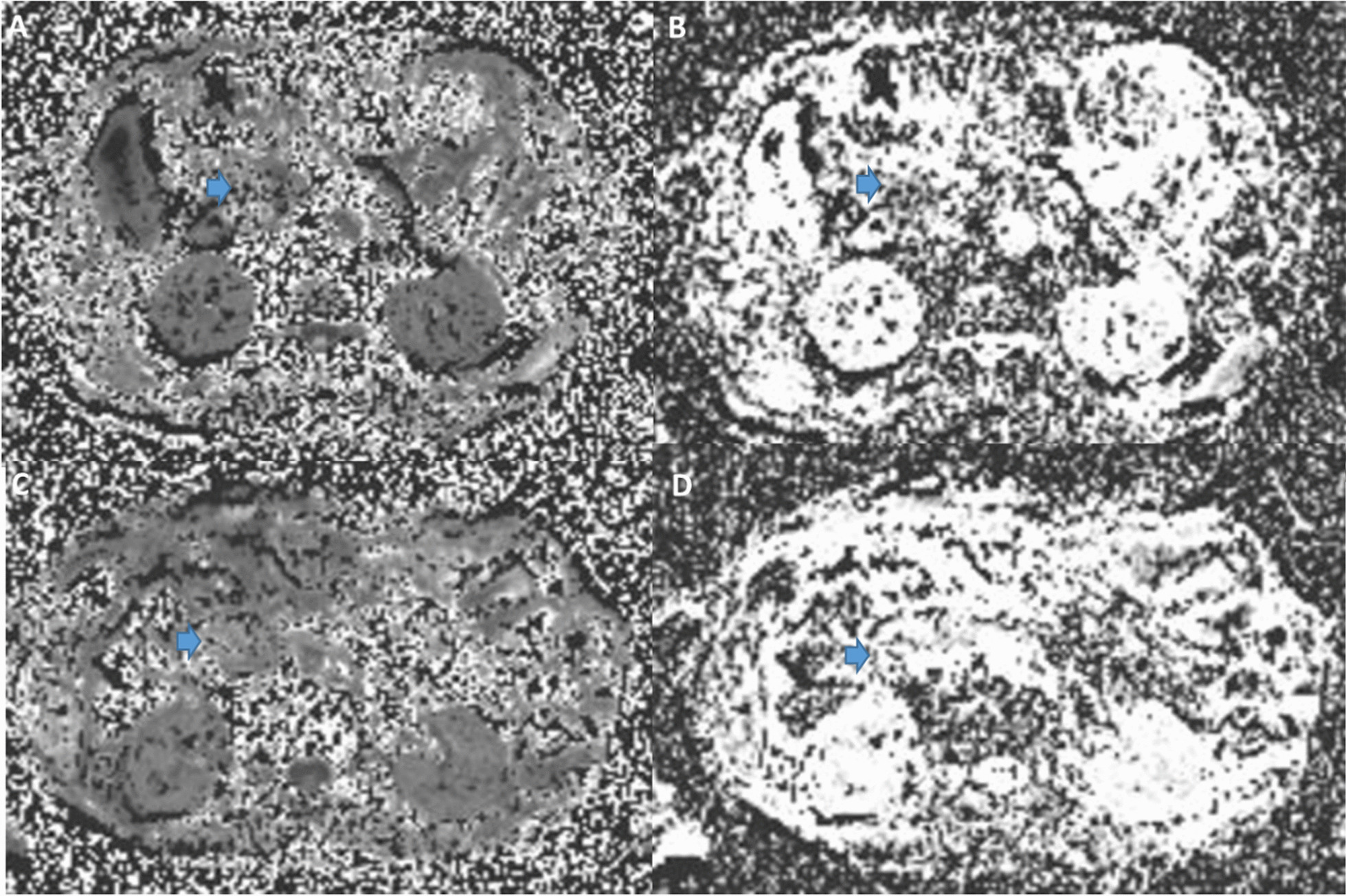
Adenocarcinoma of the pancreatic head. MK map before and after treatment (**A**, **C**); MD map before and after treatment (**B**, **D**). In MK map can be observed a reduction of signal intensity while in MD map can be observed an increase of intensity. In both cases the variation of intensity was linked to a good response after the treatment

Diagnosis of pancreatic cancer remains challenging, due to overlapping imaging features with benign lesions (Fig. 2). However, an accurate detection and characterization of lesions is required since the prognosis is connected to tumor type and grade, so as it is required a correct staging. Thus, an imaging tool that provides higher tumor conspicuity would be needed to enhance staging and clinical outcomes [102–105]. Granata et al. [106] assessed functional MRI features to differentiate pancreatic tumours, perilesional inflammatory tissue, and normal parenchyma. They used dynamic contrast-enhanced MRI (DCE-MRI), DKI, intravoxel incoherent motion (IVIM), and conventional DWI-derived parameters showing that MD by DKI, could be helpful for the differentiation of lesion to normal parenchyma and perilesional inflammation.

Shi et al. [107] assessed MRI performance in differentiating pancreatic ductal adenocarcinomas (PDACs), from solid pseudo papillary neoplasms (SPNs) and pancreatic neuroendocrine tumors (PNETs) using DKI. Considering that therapeutic strategies differ significantly between PDACs, and SPNs and PNETs, since for PDACs, aggressive surgical approaches such as the Whipple technique with extensive lymph node dissections is usually used, which entails higher post-surgical complications, it is crucial a pre-surgical staging in order to plan the more appropriate technique. The researches [107] showed that the accuracy rate with DKI for differentiating PDAC from SPNs and PNETs was higher than that of subjective diagnosis alone (*P* < 0.05) so that DKI could assist radiologists in accurately diagnosis.

Electrochemotherapy (ECT) is an interesting approach for treatment of several tumours [108–112]. This technique links the administration of drugs with electric pulses for cell membrane electroporation and it is efficacy and safety in the treatment of PDCA [113]. However, the correct assessment of this treatment is a challenge for radiologists since tumour necrosis is not associated with a dimensional change. In this scenario, the response evaluation criteria in solid tumour (RECIST) are not adequate. Granata et al. assessed Conventional DWI and DKI as tools to evaluate treatment efficacy. They showed that MD improved the diagnostic performance respect to ADC in the response assessment [114].

#### Rectal cancer

Although rectal cancer is one of the most common tumour worldwide and, though in order to identify the lesion as soon as possible, there were a widespread use of screening, however, to day, this cancer is still diagnosed in an advanced stage of the disease [115–118]. Nowadays, the standard of care in patients with locally advanced rectal cancer (LARC) is still preoperative chemoradiotherapy (pCRT) followed by total mesorectal excision [119–122], though in-patient with a complete response to treatment, it is possible to consider a “wait-and-watch” strategy.

**Fig. 3:**
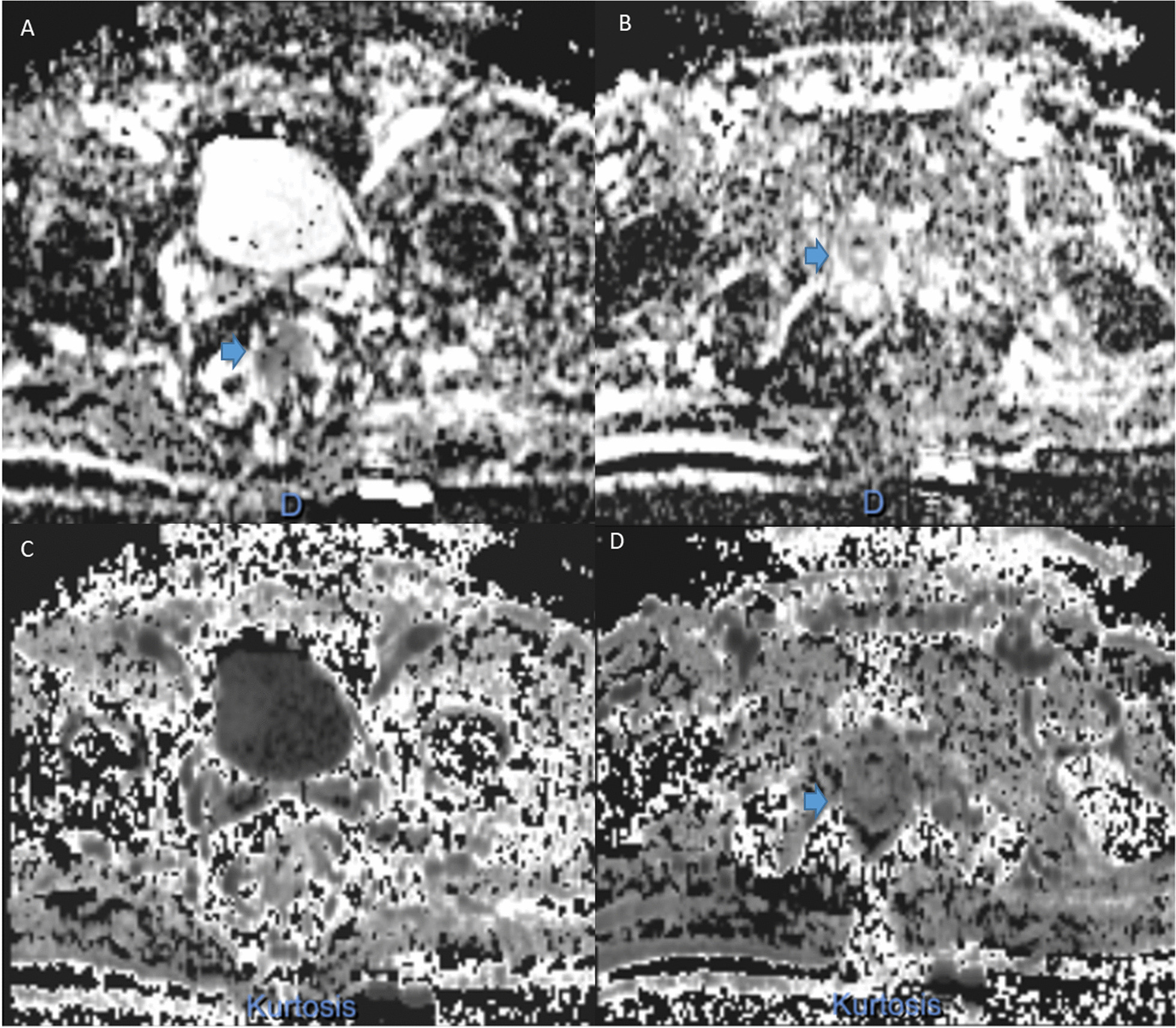
DKI-derived parameters maps pre (**A**, **C**) and post-treatment (**B**, **D**) for a responder patients (TRG 2): MK **A** and **B**, MD **C**, **D**. In MK map can be observed a reduction of signal intensity while in MD map can be observed an increase of intensity. In both cases the variation of intensity was linked to a good response after the treatment

Conventional o morphological (m)-MRI, based on T2-W sequences, is at present believed the main imaging toll for staging. However, conventional sequences have some weaknesses, particularly after pCRT. To surmount this limit, functional data have been evaluated [123–132] (Fig. 3).

The managing of rectal cancer patient is largely founded on the recurrence risk stratification. Prognostic signs involve TNM stage, histological grade, peritumonic lymphangiovascular invasion (LVI) or neural invasion, circumferential margin (CRM) involvement [128]. Zhu et al. [133] evaluated DKI (*b*-value > 2100 s/mm^2^) and conventional DWI (*b*-value > 1000 s/mm^2^) in 56 rectal cancers, evaluating the relationship between kurtosis, diffusivity, ADC with pT and pN stages and histological degrees. Kurtosis was significantly higher in tumors with nodal involvement than in those without nodal involvement. Furthermore, kurtosis was significantly higher in high-grade than low-grade tumors, while no significant differences in diffusivity or ADC were found between low- and high-grade tumors. Cui et a [134] evaluated the associations between conventional DWI (highest *b*-value 1000 s/mm^2^) and DKI (highest *b*-value 2100s/mm^2^) and plasma carcinoembryonic antigen level, pT stage, pN stage, grade tumor, peritumor LVI or neural invasion and CRM invasion in 79 patients with LARC, demonstrating that kurtosis was greater in patients with lymph node and CRM involvement, low grade lesion and presence of LVI. ADC and diffusivity were significantly correlated at stages T and N. Yu et al. [135] evaluated DKI values in lymph node involvement (85 patients with 273 lymph nodes). Dapp, Kapp, and ADC of the lymph nodes were evaluated. Median Dapp and ADC values of malignant lymph nodes were significantly higher than in benign lymph nodes, while median Kapp of malignant lymph nodes was statistically lower than in normal lymph nodes [135].

Yu et al. [136] assessed the correlation between DWI and DKI and distant metastases showing that the Dapp was significantly lower in patients with metastases [136].

Regarding the response to treatment in LARC, Yu et al. [137] evaluated DKI as a biomarker to predict the response in LARC. Researchers demonstrated that percentage change in Dapp has higher diagnostic performance for assessing response to treatment. Hu et al. [138] evaluated DKI parameters as biomarkers of complete response relative to ADC, demonstrating that MKpre and MKpost values were much lower for responder patients than for non-responders, while ADCpost and rate of change ADCs were significantly higher for responder patients. Fusco et al. [123] evaluated the tumor response to short-term radiotherapy using the standardized index of shape (SIS) by contrast magnetic resonance imaging, ADC, IVIM and DKI parameters. Promising results were obtained using a decision tree tested with all ADC, IVIM and DKI parameters.

DKI is a promising approach in evaluating LARC patients; however, the DKI must be a reproducible model. Therefore, to obtain quantitative parameters it is necessary to standardize the sequence and the model [139, 140].

#### Renal tumours

Renal cell carcinoma (RCC) is the most frequent malignant renal tumour in adults and surgical resection is the main valuable approach; other options, comprising RFA, cytoablation and even active surveillance have been employed [141–151]. In patients unfit for surgery, systemic therapy including targeted agents, immunotherapy and chemotherapy were employed to improve the overall survival (OS) [141–151]. In this context, lesion detection and identification of histologic grades has clinical significance in establishing prognosis.

Ding et al. [152] showed that DKI was a feasible tool in characterization of malignant lesions, with the MD higher, while MK lower than those of benign lesions [152]. In addition, Fu et al. [153] assessed the DKI in RCC, considering 66 patients, 13 with renal angiomyolipoma with minimal fat (RAMF) and 7 patients with renal oncocytoma (RO). MD, fractional Anisotropy (FA), MK, kurtosis anisotropy (KA) and radial kurtosis (RK) were calculated. For MD, a significant higher value was shown in RCC than the rest renal tumors. The MD values were higher for RO than for AML, while comparable MD values were found between RCC and RO. For MK, KA and RK, a significant higher value was demonstrated in AML than RCC and RO. The MK, KA and RK values were higher for RO than for RCC [153]. Zhu et al. [154] assessed the feasibility and reproducibility of diffusion kurtosis tensor imaging (DKTI) in RCC in distinguishing the subtypes of RCC and the grades of clear cell RCC (CCRCC). They found significant differences between the DKTI metrics of RCCs and contralateral renal parenchyma among the subtypes of RCC. MK and Ka values of CRCC were significantly higher than those of CCRCC and contralateral normal parenchyma (PRCC). Statistical difference of the MK, KA, RK and MD values were also obtained between CCRCC with high- and low-grades. MK values were more effective for distinguishing between low- and high- grade. These data were similar to ones of Cao et al. [155] that analysed 89 patients with histologically proven ccRCC, showing that compared to normal renal parenchyma, ADC and MD values of ccRCC decreased and MK, Ka, and Kr values increased. ADC and MD values of ccRCC decreased with the increase in pathological grade, while MK, Ka, and Kr values were increased. ADC could discriminate the grading except for G1 versus G2 while Ka and Kr the grading except for G2 versus G3 and MD and MK could discriminate G1 versus G2, G1 versus G3, G1 versus G4, G2 versus G3, G2 versus G4, and G3 versus G4. The AUC of MK was the highest [155].

Although several researches have demonstrated the potential role of DKI in the assessment of sevral prognostic features and, so to guide a precise treatment, however the potential advantage of using DKI in the kidneys remains to be fully explored.

#### Prostate cancer

In the last decade, there has been growing attention on MRI of the prostate as new imaging toll, that thanks to the association of DWI and DCE with conventional T1- and T2-W imaging, now it is possible to obtain a multiparametric MRI (mpMRI) protocols [28, 30, 156–163].

**Fig. 4:**
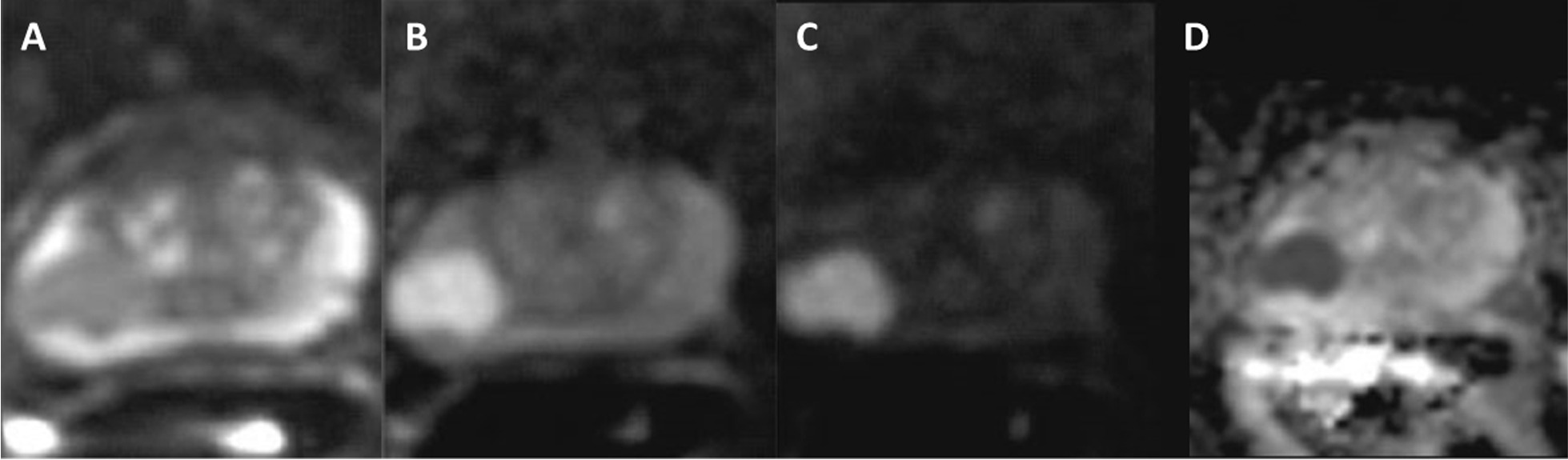
Diffusion Weigthed Images at *b* value B0 (**A**), B1000 (**B**), B2000 (**C**) and ADC map (**D**) in a prostate cancer tumor with Gleason score 7. The ADC map showed a marked narrowing of the diffusion of water molecules with a clear reduction of the signal intensity

Several researches have assessed DKI and DWI in the assessment of tumour aggressiveness (Fig. 4). However, the results have been contradictory: several studies demonstrated a better performance of DKI [164, 165], others did not prove these data [166–168]. Rosenkrantz et al. [164] showed that K values were significantly higher in both tumor than normal parenchyma and tumor with higher rather than lower Gleason scores. Furthermore, DKI showed significantly greater sensitivity than ADC to differentiate cancerous areas from benign areas in the peripheral zone (PZ). Roethke et al. [167], evaluating 55 patients with prostate cancer, did not confirm these results, showing that although K was significantly higher in areas with cancer, the ROC analysis did not show a significant difference between DKI and ADC to detect the cancer. As for the aggression, Kapp and ADC showed a comparable result.

A recent study comparing ADC and DKI in detection and characterization, evaluating 255 patients with PC [169]. The authors showed that ADC and DKI had a similar diagnostic performance, so that they concluded that there was not a clear benefit of DKI.

Therefore, the value of additional DKI remains unclear. Anyway, the present data propose that DKI could offer different but complementary information on tumour microenvironment.

#### Other fields

Few studies have evaluated the role of DKI in cervical or endometrial cancer [170–172].

Cervical cancer (UCC) was the fourth most commonly diagnosed malignancy and the fourth leading cause of cancer death among women worldwide [13, 173–178]. Cervical squamous carcinoma (CSC) is the most common pathological type of UCC, accounting for 75–80% of the total number of cervical cancer patients [170]. Poorly differentiated CSCs can easily cause local invasion and distant metastasis, influencing the choice of treatment and patient prognosis. Therefore, it is important to accurately assess the degree of CSC before treatment. Hou et al. compared the weighted imaging value for amide proton transfer (APTWI) and DKI in evaluating the histological grade of cervical squamous carcinoma (CSC) in addition to DWI [170]. They showed that the APTWI (MTRasym) parameters and MK values of G1 were significantly lower than those of G2 and those parameters of G2 were significantly lower than those of G3. The MD and ADC values of G1 were significantly higher than those of G2 and those of G2 were significantly higher than those of G3. MTRasym and MK were both positively correlated with histological grade, while MD and ADC were both negatively correlated with histological grade.

Endometrial cancer (EC) is the most common gynecological malignancy in the world. Prognosis depends on several factors, including histological grade and subtype, International Federation of Gynecology and Obstetrics (FIGO) stage, lymphovascular invasion, and lymph node metastases. The histological grade of endometrial cancer alone is a strong predictor of lymph node metastasis [130, 178–182]. Although several studies have explored the value of whole tumor histogram analysis of ADC for preoperative tumor classification of endometrial cancer, few studies have evaluated the value of DKI. Chen et al. [171] evaluated 73 patients with CE and compared Dapp, Kapp and ADC parameters between high-grade (grade 3) and low-grade (grade 1 and 2) tumors, demonstrating that the 10th percentile AUC Dapp, Kapp’s 90th percentile, and ADC The 10th percentile was superior to other parameters in distinguishing high-grade from low-grade cancers. The combination of the 10th percentile of Dapp and the 90th percentile of Kapp improved the AUC to 0.901, which was significantly higher than that of the 10th percentile of the ADC.

Yue et al. [172] compared the performance of DKI and DWI for diagnosis and histological classification of EC. They evaluated 61 EC patients and 30 patients with normal endometrium; showed that MK values for groups G0, G1, G2 and G3 gradually increased, while MD and ADC values gradually decreased. MK values had the highest diagnostic accuracy in differentiating G0 and (G1 + G2 + G3), G0 and G1, G1 and G2 and G2 and G3. MK was maximally correlated with histological grade, followed by MD and ADC [172].

## Discussion and conclusion

DKI provides more data on tissue structures than the conventional monoexponential model for *b*-values below 1000 s/mm^2^. Advances in hardware and software within modern MRI scanners now allow for ultra-high *b*-value imaging, hence the ability to apply DKI to multiple extracranial sites. Therefore, body radiologists could benefit from a better understanding of the main concepts of DKI.

Several studies have evaluated the ability of DKI in tumor characterization and tumor grade assessment. DKI parameters could help distinguish benign from malignant tissues, as several research suggests that DKI parameters outperform ADC to distinguish low- and high-grade lesions. However, these researches observe an inverse association between Kapp and ADC, raising the question of whether there is an additional advantage of DKI, given the increased technical complexity.

Additionally, in several studies the DKI sequences used were based on planar echo (EPI) acquisition, which is susceptible to motion, metal and air artefacts and prone to low SNRs and distortions, leading to low quality images for some small lesions, which may affect the accuracy of the results. Another problem is the optimal *b*-value of DKI, which remains to be explored since a publicly recognized standard has not yet been introduced, as well as the manually selected region of interest, which could affect the accuracy of some parameters.

In conclusion, DKI is still largely a research tool and few data support its routine use compared to conventional DWI in oncology. However, the technique is at a stage where it can be explored in broader clinical settings.

## Data Availability

Data are availabe at https://zenodo.org/record/6598327#.YpX2A2hBy3A.

## Abbreviations

DWI: Diffusion-weighted imaging
MRI: Magnetic resonance imaging
SI: Signal intensity
ADC: Apparent diffusion coefficient
DKI: Diffusion kurtosis imaging
MK: Kurtosis median coefficient
MD: Mean diffusion coefficient
SNR: Signal-to-noise ratio
HCC: Hepatocellular carcinoma
MVI: Microvascular invasion
STD: Standard deviation
FP: Perfusion fraction
AUC: Area under curve
IVIM: Intravoxel incoherent motion
PDACs: Pancreatic ductal adenocarcinomas
PNETs: Pancreatic neuroendocrine tumors
SPNs: Solid pseudo papillary neoplasms
ECT: Electrochemotherapy
RECIST: Response evaluation criteria in solid tumour
LARC: Locally advanced rectal cancer
pCRT: Preoperative chemoradiotherapy
LVI: Lymphangiovascular invasion
CRM: Circumferential margin
SIS: Standardized index of shape
RCC: Renal cell carcinoma
RFA: Radiofrequency ablation
OS: Overall survival
RAMF: Renal angiomyolipoma with minimal fat
RO: Renal oncocytoma
FA: Fractional anisotropy
KA: Kurtosis anisotropy
RK: Radial kurtosis
AML: Renal angiomyolipoma
DKTI: Diffusion kurtosis tensor imaging
CCRCC: Clear cell RCC
PRCC: Contralateral normal parenchyma
DCE: Dynamic contrast enhanced
mpMRI: Multiparametric MRI
UCC: Cervical cancer
CSC: Cervical squamous carcinoma
APTWI: Weighted imaging value for amide proton transfer
EC: Endometrial cancer
FIGO: International federation of gynecology and obstetrics
EPI: Echo planar imaging
